# Efficacy and safety of switching to insulin glargine 300 U/mL from 100 U/mL in Japanese patients with type 2 diabetes: A 12-month retrospective analysis

**DOI:** 10.1016/j.heliyon.2019.e01257

**Published:** 2019-02-21

**Authors:** Kazutoshi Sugiyama, Shu Meguro, Yoshifumi Saisho, Junichiro Irie, Masami Tanaka, Hiroshi Itoh

**Affiliations:** Division of Endocrinology, Metabolism, and Nephrology, Department of Internal Medicine, Keio University School of Medicine, 35 Shinanomachi, Shinjuku-ku, Tokyo 160-8582, Japan

**Keywords:** Metabolism, Evidence-based medicine

## Abstract

**Aims:**

To evaluate the efficacy and safety of switching to insulin glargine 300 U/mL (Gla-300) from insulin glargine 100 U/mL (Gla-100) in Japanese patients with type 2 diabetes (T2DM).

**Methods:**

This was a 12-month retrospective study comprising 109 patients. Primary endpoint was glycated hemoglobin (HbA1c) level at month 12. Secondary endpoints were hypoglycemia for the overall study period as well as body weight and insulin dose at month 12.

**Results:**

Similar glycemic control was achieved with mean (standard deviation) HbA1c level of 7.7 (1.1)% (61 [12] mmol/mol) at baseline and 7.7 (1.3)% (61 [14] mmol/mol) at month 12. Fewer confirmed (<3.0 mmol/L [< 54 mg/dL]) or severe hypoglycemic events were observed (0.52 vs. 0.85 events per patient-year; rate ratio 0.61; 95% confidence interval 0.38–0.97; p = 0.037), but the percent of patients experiencing ≥1 hypoglycemic event did not differ. There was no difference in confirmed (≤3.9 mmol/L [≤ 70 mg/dL]) or severe hypoglycemia and nocturnal hypoglycemia.

**Conclusions:**

In Japanese patients with T2DM who switched to Gla-300 from Gla-100, similar glycemic control was achieved with fewer confirmed (<3.0 mmol/L [< 54 mg/dL]) or severe hypoglycemic events over a 12-month period, although the absolute benefit was marginal.

## 1 Introduction

The importance of achieving a good glycemic control in order to prevent microvascular diseases in patients with type 2 diabetes (T2DM) has been well documented [1, 2, 3]. Although it can be initially achieved with lifestyle modifications and oral antihyperglycemic drugs, many patients with T2DM eventually require and benefit from insulin therapy due to its progressive nature [4]. However, insulin therapy is associated with more frequent hypoglycemia, exposing patients to an array of autonomic and neuroglycopenic symptoms [5]. Consequently, hypoglycemia is associated with a lower health-related quality of life [6] and is the major limiting factor in the glycemic management of diabetes [7].

Recently, insulin glargine 300 U/mL (Gla-300) was approved for the treatment of diabetes. It has a more constant and prolonged pharmacokinetic (PK) and pharmacodynamic (PD) profile than those of insulin glargine 100 U/mL (Gla-100) and has been expected to confer a lower hypoglycemic risk [8, 9]. The efficacy and safety of Gla-300 vs. Gla-100 were investigated in the EDITION program of randomized phase III studies, and patients treated with Gla-300 experienced fewer nocturnal hypoglycemia while achieving a similar glycemic control [10, 11, 12, 13, 14, 15, 16, 17]. However, there have been concerns that newer, even higher-priced basal insulin analogs are now being promoted despite their minor absolute benefits [18]. The aim of this study was to examine whether these results from randomized studies can be translated into real-world patients.

## 2 Materials and methods

### 2.1 Research design

This was a 12-month retrospective study using the record of patients from the Keio University Hospital (Tokyo, Japan). Clinical care was provided by diabetologists. The ethical guidelines for medical and health research involving human subjects were followed and the Keio University School of Medicine Ethical Committee approved the study protocol (No. 20130453).

Primary endpoint was glycated hemoglobin (HbA1c) level at month 12. Secondary endpoints were hypoglycemia for the overall study period as well as body weight and insulin dose at month 12. Data pertaining to HbA1c level, body weight, and insulin dose were extracted from the medical record. The day when the patients were switched from Gla-100 to Gla-300 was defined as day 0, and the baseline value was the nearest date when the relevant data were available within days -45 to 0. Days 90, 180, 270, and 360 were defined as the nearest date when the relevant data were available within days 45–134, 135–224, 225–314, and 315–404, respectively.

The hypoglycemic events recorded by the physician/nurse in the medical record and self-monitored blood glucose (SMBG) values recorded in the patient's diary were included in the analysis. Events within days -90 to -1 served as the reference compared with events within days 1–90, 91–180, 181–270, and 271–360. Hypoglycemic categories included a) confirmed (≤3.9 mmol/L [≤ 70 mg/dL]) or severe hypoglycemia (severe cognitive impairment requiring external assistance for recovery), b) confirmed (<3.0 mmol/L [< 54 mg/dL]) or severe hypoglycemia, and c) nocturnal hypoglycemia; confirmed (≤3.9 mmol/L [≤ 70 mg/dL]) or severe hypoglycemia between 00:00 and 05:59 h or in which the word “nocturnal” was present in the medical records. These hypoglycemic categories were chosen to be in line with the reported outcomes in the EDITION program.

### 2.2 Study patients

Japanese adults ≥18 years with T2DM treated with Gla-100 for ≥6 months who switched to Gla-300 during their outpatient visit between September 1, 2016 and December 31, 2016 were included. As of September 1, 2016, in-hospital prescription of Gla-100 became unavailable as a new contract with Gla-300 was implemented and virtually all patients switched from Gla-100 to Gla-300. Only six patients with T2DM switched to basal insulin other than Gla-300.

Exclusion criteria were a) change in patient's diabetes treatment regimen except insulin dose titration or switch to combination oral antihyperglycemic drugs at similar doses, b) use of premixed insulin, c) hospitalization for education on diabetes or hospitalization for more than 28 days, d) use of glucocorticoids, or e) red blood cell transfusion within 3 months prior to and 90 days after the switch. Patients with no routine visit within four months prior to the switch or after the switch and patients not performing SMBG were also excluded. If the patients met any of the above exclusion criteria for the first time after 90 days of switch, data following that date was excluded from the analysis.

### 2.3 Statistical analyses

Continuous variables were analyzed using a mixed-effects model for repeated measures (MMRM) with an unstructured covariance structure. Hypoglycemic events were analyzed using generalized estimating equations (GEE) with an unstructured correlation structure. The number of hypoglycemic events per 90 days (RR; rate ratio) was calculated using a negative binomial regression model with logarithm of the treatment period as offset, and the percent of patients experiencing ≥1 hypoglycemic event per 90 days (OR; odds ratio) was calculated using a binomial logistic regression model. A two-sided p value of less than 0.05 was considered to indicate statistical significance. All statistical analyses were performed using IBM SPSS Statistics for Windows Version 25.0 (IBM Corp., Armonk, NY, USA).

## 3 Results

### 3.1 Study patients

The study included 109 patients with T2DM. Their baseline characteristics are presented in Table 1. Records of SMBG diary were available for 81 (74.3%) patients, and among those patients, the median (interquartile range) number of SMBG per day was 1.8 (1.0–2.0).

**Table 1:** Baseline characteristics of study patients. Data are n (%), mean (standard deviation), or median (interquartile range). BMI, body mass index; DPP-IV, dipeptidyl peptidase-4; GLP-1, glucagon-like peptide-1; HbA1c, glycated hemoglobin; SGLT2, sodium-glucose cotransporter 2.

	N = 109
Age (years)	67.3 (12.3)
Male	73 (67.0)
Duration of type 2 diabetes (years)	23.0 (11.3)
Duration of insulin use (years)	11.1 (8.0)
Body weight (kg)	69.0 (16.7)
BMI (kg/m^2^)	25.9 (5.8)
Random blood glucose (mmol/L)	9.1 (2.9)
(mg/dL)	163.3 (53.0)
HbA1c (%)	7.7 (1.1)
(mmol/mol)	61 (12)
Dose of basal insulin (U/d)	12.4 (6.0–16.5)
Dose of mealtime insulin (U/d)	22.5 (14.0–26.0)
Antihyperglycemic drugs	
Mealtime insulin	72 (66.1)
DPP-IV inhibitors	63 (57.8)
Biguanides	30 (27.5)
Glinides	20 (18.3)
α-glucosidase inhibitors	19 (17.4)
SGLT2 inhibitors	15 (13.8)
GLP-1 receptor agonists	8 (7.3)
Sulfonylureas	7 (6.4)
Thiazolidinediones	7 (6.4)

### 3.2 Change in insulin dose

**Fig. 1:**
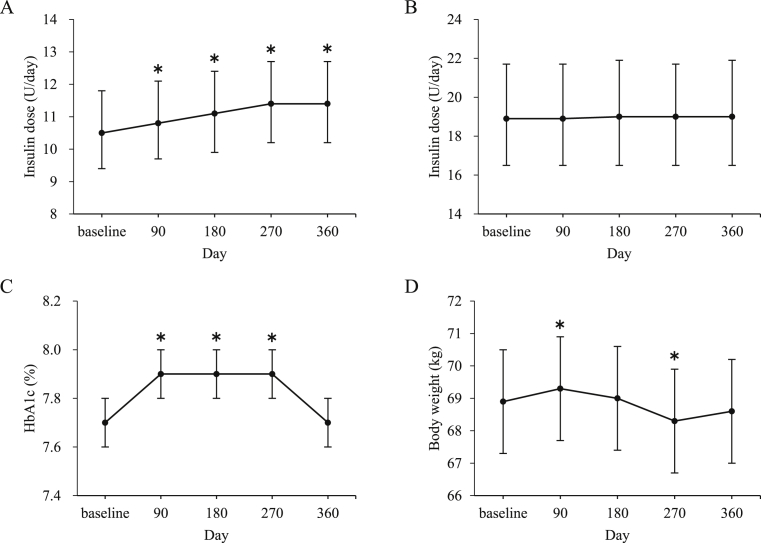
Insulin dose, glycated hemoglobin (HbA1c) level, and body weight of patients with type 2 diabetes during the 12-month study period. (A) Basal insulin dose. (B) Mealtime insulin dose. (C) HbA1c level. (D) Body weight. Data are shown as mean ±95% confidence interval of basal and mealtime insulin dose, and mean ± standard error of HbA1c level and body weight. *p < 0.05 in comparison with that of the baseline.

Mean (95% confidence interval [CI]) basal insulin dose increased from 10.5 (9.4–11.8) U/d at baseline to 10.8 (9.7–12.1) U/d at day 90 (p < 0.001), 11.1 (9.9–12.4) U/d at day 180 (p < 0.001), 11.4 (10.2–12.7) U/d at day 270 (p < 0.001), and 11.4 (10.2–12.7) U/d at day 360 (p = 0.001) (Fig. 1A). The mean (95% CI) mealtime insulin dose did not differ from the baseline of 18.9 (16.5–21.7) U/d throughout the study period (Fig. 1B).

### 3.3 Glycemic control

The mean (SD) HbA1c level increased from the baseline of 7.7 (1.1)% (61 [12] mmol/mol) to 7.9 (1.1)% (63 [12] mmol/mol) at day 90 (p = 0.003), 7.9 (1.2)% (63 [13] mmol/mol) at day 180 (p = 0.013), and 7.9 (1.3)% (63 [14] mmol/mol) at day 270 (p = 0.042), but it did not differ significantly at day 360 with 7.7 (1.3) % (61 [14] mmol/mol) (Fig. 1C).

### 3.4 Confirmed (≤3.9 mmol/L [≤ 70 mg/dL]) or severe hypoglycemia

**Fig. 2:**
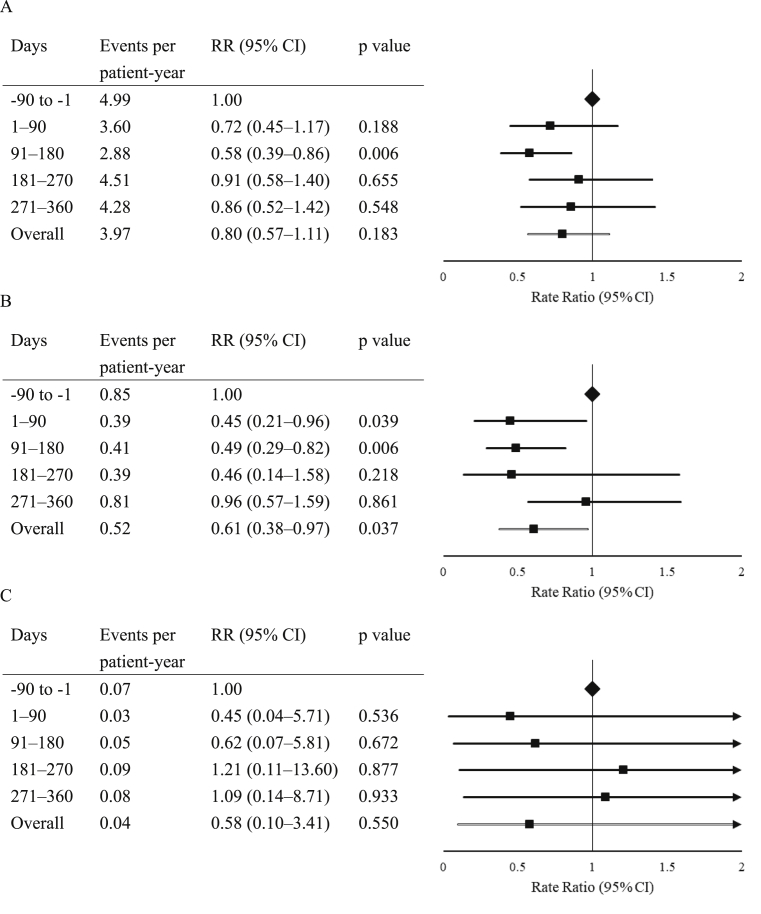
Average number of hypoglycemic events per patient-year in patients with type 2 diabetes during the 12-month study period. (A) Confirmed (≤3.9 mmol/L [≤7 0 mg/dL]) or severe hypoglycemia. (B) Confirmed (<3.0 mmol/L [< 54 mg/dL]) or severe hypoglycemia. (C) Nocturnal hypoglycemia. CI, confidence interval; RR, rate ratio. Right arrow represents RR ≥ 2.0.

Patients experienced on an average 4.99 confirmed (≤3.9 mmol/L [≤ 70 mg/dL]) or severe hypoglycemic events per patient-year at baseline (Fig. 2A). It was significantly fewer at days 91–180 (RR 0.58; 95% CI 0.39–0.86; p = 0.006) but did not differ throughout the remaining study period. At baseline, 25% of the patients experienced ≥1 hypoglycemic event, and there was no difference throughout the study period (Table 2A).

**Table 2:** Percent of patients experiencing ≥1 hypoglycemic event every 90 days. (A) Confirmed (≤3.9 mmol/L [≤ 70 mg/dL]) or severe hypoglycemia. (B) Confirmed (<3.0 mmol/L [< 54 mg/dL]) or severe hypoglycemia. (C) Nocturnal hypoglycemia. CI, confidence interval; OR, odds ratio.

Day	A		B		C	
	% of patients	OR (95% CI)	% of Patients	OR (95% CI)	% of patients	OR (95% CI)
-90 to -1	25	1.00	6	1.00	2	1.00
1–90	27	1.10 (0.74–1.64)	7	1.15 (0.45–2.93)	1	0.50 (0.04–5.63)
91–180	25	1.01 (0.65–1.54)	6	0.88 (0.38–2.04)	1	0.61 (0.06–6.88)
181–270	27	1.13 (0.70–1.81)	5	0.78 (0.22–2.72)	1	0.70 (0.06–7.72)
271–360	34	1.59 (0.95–2.67)	8	1.23 (0.49–3.06)	2	0.84 (0.08–9.02)

### 3.5 Confirmed (<3.0 mmol/L [< 54 mg/dL]) or severe hypoglycemia

Patients experienced on an average 0.85 confirmed (<3.0 mmol/L [< 54 mg/dL]) or severe hypoglycemic events per patient-year at baseline (Fig. 2B). It was significantly fewer on days 1–90 (RR 0.45; 95% CI 0.21–0.96; p = 0.039) and days 91–180 (RR 0.49; 95% CI 0.29–0.82; p = 0.006) but was not significant on days 181–270 and 271–360. Overall, there was a 39% decrease in hypoglycemic events (RR 0.61; 95% CI 0.38–0.97; p = 0.037). At baseline, 6% of the patients experienced ≥1 hypoglycemic event, and there was no difference throughout the study period (Table 2B).

### 3.6 Nocturnal hypoglycemia

Patients experienced on an average 0.07 nocturnal hypoglycemic events per patient-year at baseline, and there was no difference throughout the study period (Fig. 2C). At baseline, 2% of the patients experienced ≥1 nocturnal hypoglycemic event, and there was no difference throughout the study period (Table 2C).

### 3.7 Body weight

The mean (SD) body weight significantly increased from the baseline of 68.9 (16.7) kg to 69.3 (16.9) kg at day 90 and decreased to 68.3 (17.1) kg at day 270, but did not differ at days 180 and 360, with 69.0 (17.1) and 68.6 (17.1) kg, respectively (Fig. 1D).

## 4 Discussion

In this observational retrospective study, Japanese patients with T2DM who switched to Gla-300 from Gla-100 were examined for 12 months. A significant increase in basal insulin dose was observed, but it was not associated with weight gain at day 360. The HbA1c level did not differ on day 360, but an increase was observed at days 90, 180, and 270. Seasonal fluctuations in HbA1c level are known, with the highest HbA1c level during winter-spring and lowest during summer-autumn [19, 20]. As days 90 and 180 correspond to winter-spring, seasonal fluctuations might have accounted for the increase in HbA1c level during the study period. A decrease in annualized rates for confirmed (<3.0 mmol/L [< 54 mg/dL]) or severe hypoglycemia was observed, while the percent of patients experiencing ≥1 hypoglycemic event did not differ, suggesting a benefit in patients experiencing multiple events. There was no difference in confirmed (≤3.9 mmol/L [≤ 70 mg/dL]) or severe hypoglycemia and nocturnal hypoglycemia.

A characteristic feature of the EDITION program was the reduction in nocturnal hypoglycemic events, which is in contrast with the results of our study. This discrepancy might be in part due to the low number of nocturnal hypoglycemic events observed. For example, the number of nocturnal hypoglycemic events per patient-year in EDITION JP 2 [11] was 2.09, compared with 0.04 in our study.

Several reasons might have accounted for the low number of nocturnal hypoglycemic events. First, it is difficult to detect nocturnal hypoglycemic events only from symptoms, especially in the elderly with impaired awareness of hypoglycemia [21, 22]. In a study evaluating hypoglycemia using blinded continuous glucose monitoring in elderly patients, 69% of the patients experienced at least one nocturnal hypoglycemic event, but all events were unrecognized [23]. Second, even when hypoglycemic events are recognized, patients can be reluctant to report hypoglycemic events in a real-world setting [24]. For example, in a survey of Japanese patients with diabetes, 181 patients reported of experiencing at least one nocturnal hypoglycemic event in the past 3 months, but only 33% of the patients contacted a healthcare professional after experiencing the event [25]. Third, although we excluded patients not performing SMBG, and records of SMBG diary were available for 74.3% of the patients in addition to the physician/nurse medical record, the retrospective nature is a limiting factor in the collection of data.

Another potential factor contributing to the low number of nocturnal hypoglycemic events might have been the different insulin titration strategies used. In our study, it was at the treating physician's discretion and the mean fasting SMBG value (only a reference value as data was available for roughly 70% of the patients) was 7.8 mmol/L (139.8 mg/dL) at baseline and 8.0 mmol/L (144.2 mg/dL) on day 360. On the contrary, basal insulin dose was titrated to achieve a fasting SMBG value of 4.4–5.5 mmol/L (80–100 mg/dL) in the EDITION program with achieved mean values of 6.4–7.1 mmol/L (114.4–126.9 mg/dL) at month 6. The higher fasting SMBG value observed in our study probably reflects the fact that the study population was older with longer duration of diabetes. When targeting a more realistic fasting SMBG value individualized for each patient, the differences in the PK/PD profile of Gla-300 and Gla-100 might not be relevant to obtain clinical benefits. In the SENIOR study [26], which prospectively compared Gla-300 vs. Gla-100 in elderly patients (≥65 years) with T2DM, a higher fasting SMBG target of 5.0–7.2 mmol/L (90–130 mg/dL) was used, and there was no difference in the incidence and rate of hypoglycemia.

The strengths of the present study include the following. First, the risk of selection bias was very low as virtually all patients using Gla-100 switched to Gla-300. Second, we were able to follow the patients for a 12-month period, which enabled us to account for seasonal variances.

The present study had some limitations along with its observational retrospective nature. First, there was no comparison arm. Second, we were unable to capture hypoglycemic events not reported by patients as discussed above. Third, roughly 35% of the patients met the exclusion criteria before month 12, and the number of patients with measured HbA1c values was 109, 107, 97, 78, and 71 on days 0, 90, 180, 270, and 360, respectively. 31 patients had a change in their diabetes treatment regimen, 3 patients were hospitalized for education on diabetes, 2 patients were referred to a primary care clinic, 1 patient was hospitalized for more than 28 days due to septic arthritis, and 1 patient died. Fourth, mealtime insulin accounted for roughly two-thirds of the total daily insulin dose. Although basal insulin requirements in Japanese patients with type 1 diabetes has been reported to be ∼30% of the total daily insulin dose, which is in contrast with Western countries where basal insulin requirements are ∼50% of the total daily insulin dose [27], it was difficult to assign a hypoglycemia to basal or mealtime insulin. Finally, this study only included Japanese patients who visited a single university hospital in Tokyo where clinical care was provided by diabetologists. Therefore, the results might not be applicable to patients with diabetes in the primary care setting and other ethnic groups.

In conclusion, in Japanese patients with T2DM who switched to Gla-300 from Gla-100, similar glycemic control was achieved with fewer confirmed (<3.0 mmol/L [< 54 mg/dL]) or severe hypoglycemic events during the 12-month period, although the absolute benefit was marginal. There was no difference in confirmed (≤3.9 mmol/L [≤ 70 mg/dL]) or severe hypoglycemia and nocturnal hypoglycemia.

## Declarations

### Author contribution statement

Kazutoshi Sugiyama: Conceived and designed the experiments; Performed the experiments; Analyzed and interpreted the data; Wrote the paper.

Shu Meguro: Conceived and designed the experiments; Analyzed and interpreted the data; Wrote the paper.

Yoshifumi Saisho, Junichiro Irie, Masami Tanaka, Hiroshi Itoh: Analyzed and interpreted the data.

### Funding statement

This research did not receive any specific grant from funding agencies in the public, commercial, or not-for-profit sectors.

### Competing interest statement

The authors declare the following conflict of interests:

Kazutoshi Sugiyama; no conflicts of interest.

Shu Meguro; personal fees from Takeda Pharmaceutical Co. Ltd., Japan, Nippon Boehringer Ingelheim Co. Ltd., Japan and AstraZeneca K.K., Japan outside the submitted work.

Yoshifumi Saisho; grants and personal fees from AstraZeneca K.K., Japan personal fees from Takeda Pharmaceutical Co. Ltd., Japan and Nippon Boehringer Ingelheim Co. Ltd., Japan outside the submitted work.

Junichiro Irie; personal fees from Takeda Pharmaceutical Co. Ltd., Japan outside the submitted work.

Masami Tanaka; personal fees from Novo Nordisk Pharma Ltd., Japan, Takeda Pharmaceutical Co. Ltd., Japan and Novartis Pharma Ltd., Japan outside the submitted work.

Hiroshi Itoh; grants and personal fees from Takeda Pharmaceutical Co. Ltd., Japan, Nippon Boehringer Ingelheim Co. Ltd., Japan, Daiichi Sankyo Co. Ltd., Japan, MSD K.K., Japan, Mitsubishi Tanabe Pharma Corporation, Japan, Shionogi & Co. Ltd., Japan and Taisho Toyama Pharmaceutical Co. Ltd., Japan grants from Sumitomo Dainippon Pharma Co. Ltd., Japan, Astellas Pharma Inc., Japan, Kyowa Hakko Kirin Co. Ltd., Japan, Teijin Pharma Ltd., Japan, Mochida Pharmaceutical Co. Ltd., Japan, Ono Pharmaceutical Co. Ltd., Japan, Chugai Pharmaceutical Co. Ltd., Japan, Eli Lilly Japan K.K., Japan, Sanofi K.K., Japan, Johnson & Johnson K.K., Japan and Abbott Japan Co. Ltd., Japan personal fees from Nipro Corporation, Japan, SBI Pharmaceuticals Co. Ltd., Japan outside the submitted work.

### Additional information

No additional information is available for this paper.
